# A 3-year retrospective analysis of canine intestinal parasites: fecal testing positivity by age, U.S. geographical region and reason for veterinary visit

**DOI:** 10.1186/s13071-021-04678-6

**Published:** 2021-03-20

**Authors:** Sarah Sweet, Evan Hegarty, Donald J. McCrann, Michael Coyne, Dave Kincaid, Donald Szlosek

**Affiliations:** IDEXX Laboratories, Inc., One IDEXX Drive, Westbrook, ME USA

**Keywords:** Ascarids, Canine, Coproantigen, Dog, Fecal, Flotation, *Giardia*, Hookworms, Parasitism, Whipworms

## Abstract

**Background:**

Canine life stage is a key factor in parasite prevalence as clinical signs associated with parasitism are more common in pups. In adult dogs, health status and geographical region may also play a role in parasite prevalence. The purpose of this study was to evaluate fecal test results using zinc sulfate flotation by centrifugation combined with fecal antigen testing for hookworms (*Ancylostoma* spp. *Uncinaria stenocephala*), ascarids (*Toxocara canis, Toxascaris* spp.*, Baylisascaris* spp.) and whipworms (*Trichuris vulpis*) sorted by age, geographical region and veterinary visit type.

**Methods:**

A retrospective sample of intestinal parasite panels submitted to IDEXX Laboratories from 1,626,104 individual dogs were selected from the continental USA from 1 January 2017 to 31 December 2019. These data contain results from fecal exams performed using zinc sulfate flotation by centrifugation paired with coproantigen immunoassay results for hookworms, ascarids, whipworms and *Giardia* (Fecal Dx® with *Giardia* coproantigen immunoassay plate). For paired testing, if either the coproantigen assay or flotation test was positive, the sample was considered to be positive. Data were summarized by age category, U.S. Census Bureau geographical region (Northeast, South, Midwest, West) and veterinary visit type. Visit types were subdivided into Wellness Visits and Other Clinical Visits in which a fecal sample was submitted.

**Results:**

In dogs presenting for either Wellness Visits or Other Clinical Visits in which *Giardia* testing was included, *Giardia* had the highest positivity (combined results for microscopy and coproantigen: 12.2 and 10.8%, respectively), followed by hookworms (combined microscopy and coproantigen: 4.1 and 4.2%, respectively), ascarids (combined microscopy and coproantigen: 2.5 and 1.7%, respectively) and whipworms (combined microscopy and coproantigen: 1.1 and 1.4%, respectively). When all test results were pooled together, pups aged 2–6 months were observed to have the highest proportion of positive results by either microscopy or coproantigen immunoassay regardless of clinical visit type. Parasite positivity varied by geographical region. Regardless of visit type, age or geographical region, the coproantigen method was observed to find a higher proportion of positive test results than microscopy in *Giardia*, ascarids, hookworms and whipworms.

**Conclusions:**

The Fecal Dx® coproantigen immunoassay combined with the zinc sulfate flotation by centrifugation method uncovers a higher number of positive hookworm, ascarid and whipworm infections than zinc sulfate flotation alone in both pups and adult dogs across all geographical regions of the USA regardless of visit type.
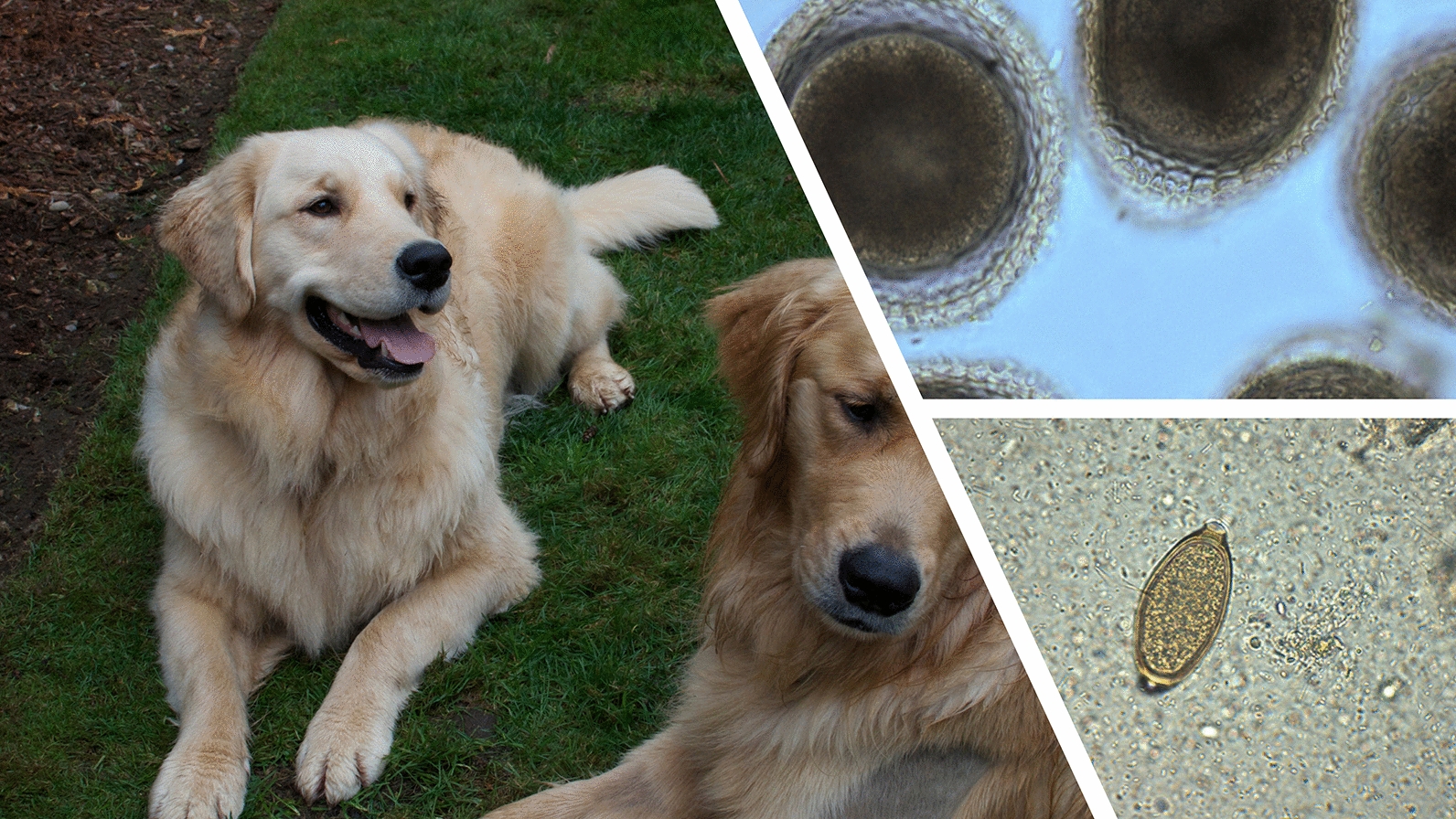

**Supplementary Information:**

The online version contains supplementary material available at 10.1186/s13071-021-04678-6.

## Background

Accurate estimation of parasite prevalence is essential for veterinarians to understand the risk of infection. A recently published study highlighted the prevalence of common intestinal parasites even in well-cared dogs [1]. For the protection of people and pets, fecal exams are recommended as part of the annual or semi-annual canine wellness visit. The Companion Animal Parasite Council (CAPC) recommends that pups receive fecal testing two to four times during their first year of life, as canine life stage has been shown to be a key factor in parasite prevalence [2]. Clinical signs associated with certain parasites (e.g. *Cystoisospora*, *Ancylostoma,* ascarids, *Giardia*) have been observed to be more common in pups [3, 4]. Fecal testing should also be conducted on any dog presenting with clinical signs consistent with intestinal parasitism, including dogs with intermittent or persistent diarrhea (CAPC guidelines) [2].

Historically, the prevalence of canine intestinal parasites across the USA has been estimated using fecal flotation and sedimentation methods [5–9]. While fecal flotation is still the most common method for the detection of intestinal parasites, microplate enzyme-linked immunosorbent assays (ELISA) for parasite-specific antigen in feces (coproantigen assay) have been shown to increase the ability to detect infections and identify cases in which egg observations may be spurious as a result of coprophagy [10]. In addition, the CAPC states that fecal tests for specific coproantigens combined with flotation have been shown to optimize results when few to no eggs are recovered or with single sex or immature worm infections [2].

The combined use of flotation with coproantigen immunoassay should provide a more representative image of the total positive tests by life stage and region in the USA. The aims of this study are: (i) to report the proportion of fecal samples testing positive for intestinal parasites stratified by life stage, geographical region and veterinary visit type; and (2) to describe differences in positivity between these two diagnostic methods (zinc sulfate centrifugation and coproantigen immunoassay).

## Methods

Test results ordered by veterinary practices in the USA were collected from samples submitted to a national commercial reference laboratory using proprietary software (IDEXX Laboratories, Inc. Westbrook, ME, USA) from 1 January 2017 to 31 December 2019. Samples from dogs, aged 2 months to 25 years, tested for hookworms (*Ancylostoma* spp*.*, *Uncinaria stenocephala*), ascarids (*Toxocara* spp*.*, *Toxascaris* spp*.*, *Baylisascaris* spp*.*) and whipworms (*Trichuris vulpis*) with the zinc sulfate flotation by centrifugation method and coproantigen immunoassay on the same visit were used as study material. Complete paired testing of flotation and coproantigen was not required for *Giardia* spp. A single centrifugation for 5 min at 500 *g* was performed using zinc sulfate flotation solution (specific gravity: 1.24), then read at 100× and 400× magnification following standard methods [11]. Coproantigen immunoassays (Fecal Dx® immunoassay plate; IDEXX Laboratories, Inc.) utilize unique capture and detection antibodies developed against recombinantly expressed proteins of ascarids (*Toxocara* spp.*, Toxascaris* spp*., Baylisascaris* spp*.*), hookworms (*Ancylostoma* spp., *Uncinaria* spp.) and whipworms (*Trichuris vulpis*) [11–13]. The coproantigen immunoassay has > 98% specificity for these three parasites. Test results for the presence of *Cystoisospora* spp.,* Eimeria* spp. and tapeworms (cestodes) (*Dipylidium* spp*.*, *Moniezia* spp*.*, *Anoplocephala* spp*.*, *Spirometra* spp., *Taenia* spp*.*, *Mesocestoides* spp*.*, *Diphyllobothrium* spp., *Echinococcus* spp*.*, *Hymenolepis* spp., *Paranoplocephala* spp., *Cittotaenia* spp.) were only available* via* zinc sulfate centrifugal flotation.

For inclusion in the study, the age of the dog and geographical location of the clinic that provided the sample were required. For dogs with multiple samples within the dataset, only the first sample for an individual dog and samples that had a second test 180 days following the first were included in the analysis. Only samples from clinical visits were included; samples from visits that were classified as retail, grooming or boarding were excluded. Clinical visits were subdivided into Wellness Visits and Other Clinical Visits. Wellness Visits were visits to the veterinarian for the purpose of annual exams, vaccinations or routine check-ups. Other Clinical Visits were defined as a newly identified clinical condition, monitoring of an existing clinical condition or for procedures such as surgery or dental prophylaxis.

All data analyses were conducted using R version 3.5.3. Data analysis was done with the tidyverse and multiple helper function within the following packages: data.table, magrittr, here, Hmisc, ggplot2, gridExtra, ggpubr, extrafont [14–20]. Summary statistics were reported as the percentage of positive results of all test results. Confidence intervals were calculated using the binomial exact method. For regional analysis of parasite positivity, U.S. Census Bureau regions (Northeast, South, Midwest and West) were used, as these align with major geographical and demographical differences that could theoretically influence parasite prevalence. Age was segmented into seven life stage categories (≥ 2–6 months, ≥ 7–12 months, > 1–2 years, ≥ 3–5 years, ≥ 6–8 years, ≥ 9–13 years and ≥ 14 years). Statistical comparisons between methods were analyzed using the two-sided Pearson’s Chi-squared 2-sample test for equality of proportions with continuity correction.

## Results

The total study population included in the analysis consisted of 1,903,852 fecal tests from canines; *Giardia* coproantigen testing was not a criterion for inclusion. Of these 1,903,852 canine fecal tests: (i) 1,626,104 were for individual dogs, with 64.9% (*n* = 1,236,448) coming from Wellness Visits and 35.1% (*n* = 667,404) coming from Other Clinical Visits; (ii) 50.1% (*n* = 953,660) included a *Giardia* coproantigen test, involving 832,446 individual dogs, of which 57.8% (*n* = 481,137) were from Wellness Visits and 42.2% (*n* = 351,309) were from Other Clinical Visits. A complete analysis of pooled wellness and other clinical visits samples can be found in the Electronic Supplementary Material (Additional file 1: Table S1; Additional file 2: Table S2; Additonal file 3: Figure S1; Additional file 4: Table S3; Additional file 5: Table S4; Additional file 6: Table S5; Additional file 7: Figure S2; Additional file 8: Figure S3; Additional file 9: Table S6).

In results from dogs presenting for a Wellness Visit in which *Giardia* testing was included, *Giardia* had the highest positivity (combined microscopy and coproantigen: 12.2%), followed by hookworms (combined microscopy and coproantigen: 4.1%), ascarids (combined microscopy and coproantigen: 2.5%), *Eimeria* spp. (microscopy: 1.8%), *Cystoisospora* (microscopy: 1.6%), whipworms (combined microscopy and antigen: 1.1%) and tapeworms (microscopy: 0.2%), (Table 1). In results from dogs presenting for Other Clinical Visits in which *Giardia* testing was included, *Giardia* also had the highest positivity (combined microscopy and coproantigen: 10.8%), followed by hookworms (combined microscopy and coproantigen: 4.2%), ascarids (combined microscopy and coproantigen: 1.7%), *Eimeria* spp. (microscopy: 1.4%), *Cystoisospora* (microscopy: 1.4%), whipworms (combined microscopy and coproantigen: 1.4%) and tapeworms (microscopy: 0.3%, Table 1). A higher proportion of positive test results was observed when coproantigen and centrifugation results were combined than with either centrifugation or coproantigen assay alone (Table 1). Age-group differences by clinical exam type were observed. Pups aged 2–6-months comprised 18.5% (*n* = 228,453) of the Wellness Visits population and only 8.9% (*n* = 59,114) of the Other Clinical Visits population (Additional file 1: Table S1). In samples from both the Wellness Visits and Other Clinical Visits, the coproantigen method found a higher proportion of positive test results than microscopy for *Giardia*, ascarids, hookworms and whipworms (Table 1).

**Table 1:** Proportion of dogs with a positive test result for an intestinal parasite by the centrifugation method or coproantigen assay during Wellness Visits or Other Clinical Visits

Parasite	Wellness Visit			Other Clinical Visits		
	Centrifugation (95% CI)	Coproantigen immunoassay (95% CI)	Combined^a^ (95% CI)	Centrifugation (95% CI)	Coproantigen immunoassay (95% CI)	Combined^a^ (95% CI)
*Giardia*	4.1% (4.0–4.1)	12% (12.0–12.1)	12.2% (12.1–12.2)	3.5% (3.4–3.6)	10.6% (10.5–10.7)	10.8% (10.7–10.9)
Hookworm	2.2% (2.2–2.2)	3.5% (3.5–3.6)	4.1% (4.1–4.1)	2.3% (2.3–2.3)	3.6% (3.6–3.7)	4.2% (4.1–4.2)
Ascarid	1.9% (1.9–1.9)	2.1% (2.1–2.1)	2.5% (2.5–2.5)	1.3% (1.2–1.3)	1.4% (1.4–1.4)	1.7% (1.7–1.7)
*Eimeria*	1.8% (1.8–1.9)			1.4% (1.4–1.5)		
*Cystoisospora*	1.6% (1.66–1.6)			1.4% (1.4–1.4)		
Whipworm	0.6% (0.6–0.7)	0.8% (0.8–0.9)	1.1% (1.1–1.1)	0.9% (0.9–0.9)	1.1% (1.1–1.1)	1.4% (1.3–1.4)
Tapeworm	0.2% (0.2–0.2)			0.3% (0.3–0.3)		

Values are presented as the percentage test positivity, with the 95% confidence interval (CI) in parenthesis

^a^Combined values represent positive results by either the coproantigen immunoassay or the centrifugation method

**Fig. 1:**
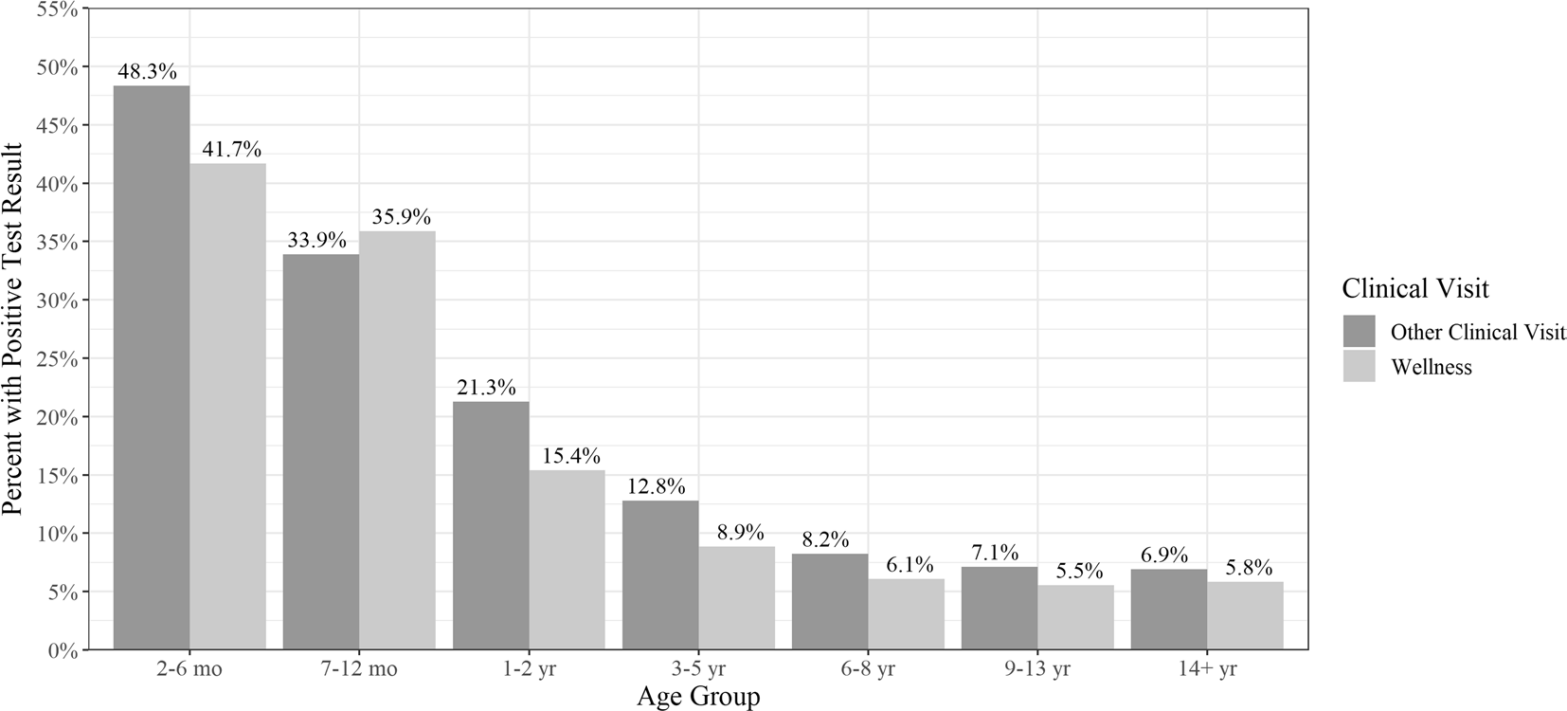
Proportion of dogs with a positive test result for any intestinal parasite by either the centrifugation method or coproantigen assay by age category for Wellness Visits (light gray) or Other Clinical Visits (dark gray). Parasites included: *Giardia*, hookworm, ascarid, whipworm, *Eimeria*, *Cystoisospora* and tapeworm

**Fig. 2:**
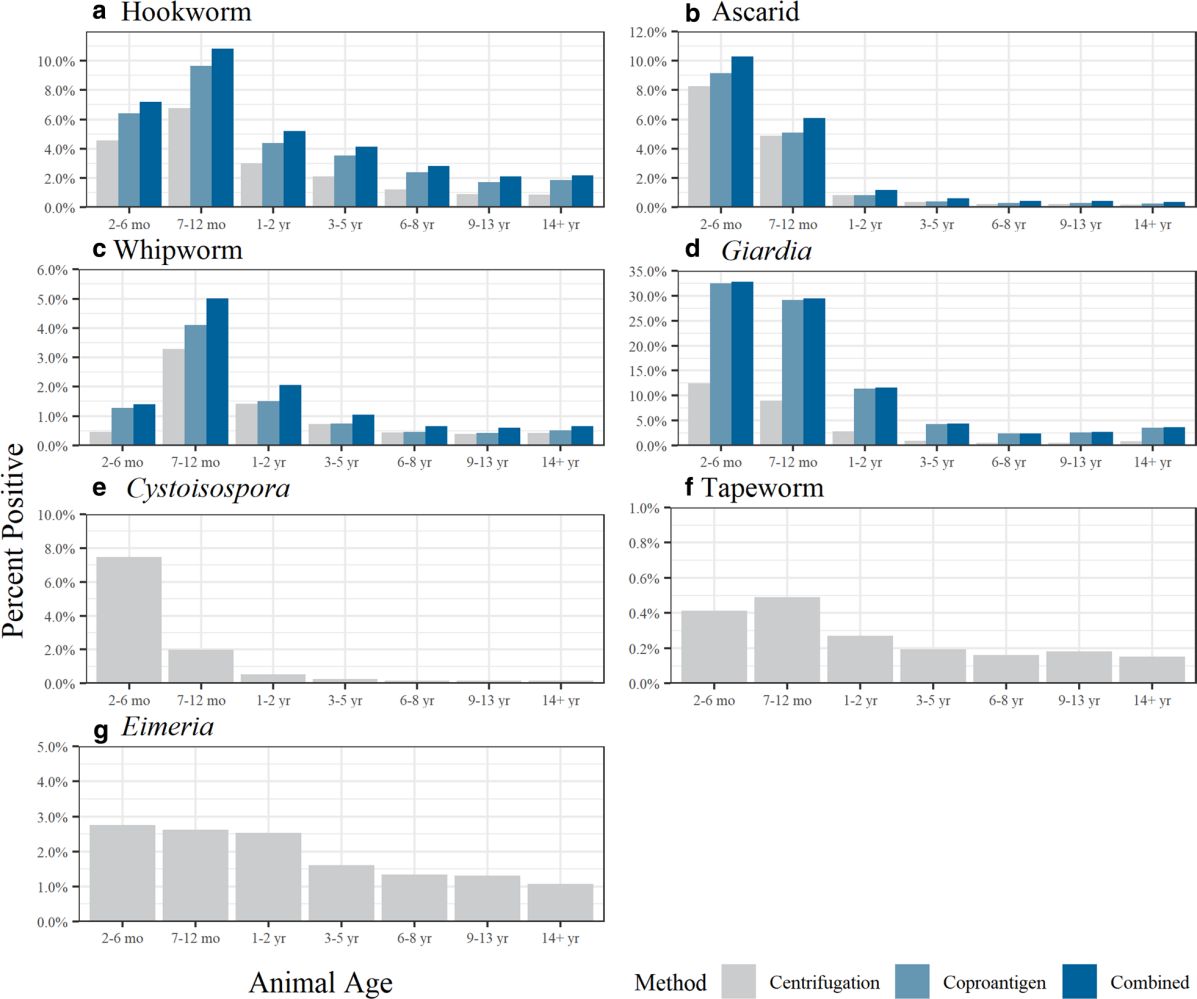
Proportion of dogs classified as Wellness Visit with positive test results for intestinal parasite by the centrifugation method and coproantigen assay by age category. Combined refers to positive results by either the coproantigen immunoassay or the centrifugation method

**Fig. 3:**
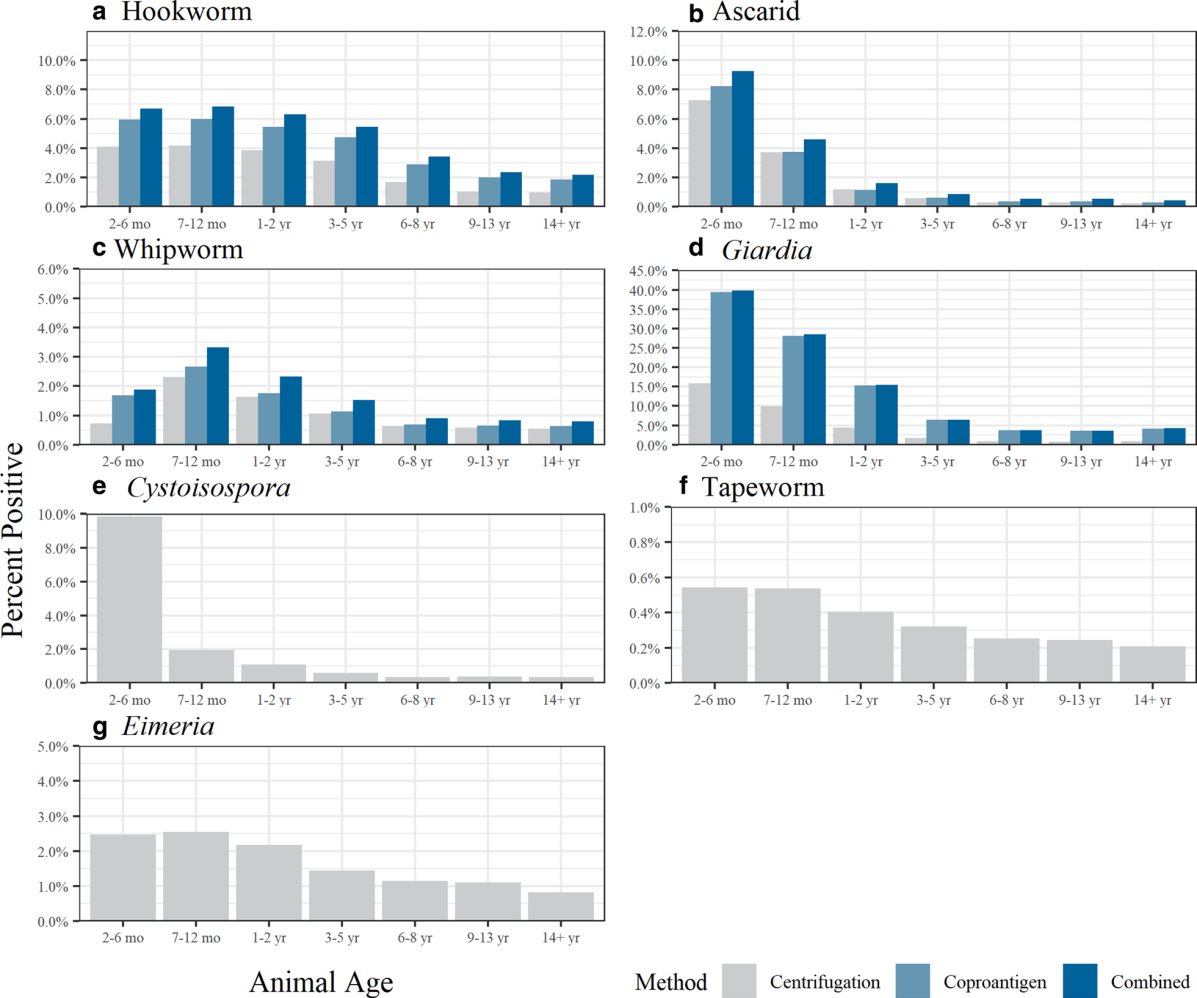
Proportion of dogs classified as Other Clinical Visits with positive test results for intestinal parasite by the centrifugation method and coproantigen method by age category. Combined refers to positive results by either the coproantigen immunoassay or the centrifugation method

When pooled together, pups in the age category 2–6-months were observed to have the highest proportion of positive test results by either the centrifugation method or coproantigen assay regardless of veterinary visit type (Fig. 1; all comparisons Pearson Chi-square *P* value < 0.001). Regardless of visit classification, samples from 2- to 6-month-old pups were found to have the highest proportion of positive test results by both the centrifugation and coproantigen compared to samples from other age categories for *Giardia*, *Cystoisospora* and ascarids (Figs. 2, 3; all comparisons Pearson Chi-square *P* value < 0.001). Samples from 7- to 12-month-old pups were found to have the highest proportion of positive test results for hookworms and whipworms by both centrifugation and coproantigen (Figs. 2, 3). In healthy adult dogs aged > 2 years, *Eimeria* spp. was the most common organism identified by centrifugation (Additional file 2: Table S2). Regardless of age or veterinary visit type, the coproantigen assay was observed find a higher proportion of positive test results than microscopy for *Giardia*, ascarids, hookworms and whipworms (Figs. 2, 3; all comparisons Pearson Chi-square *P* value < 0.001).

**Fig. 4:**
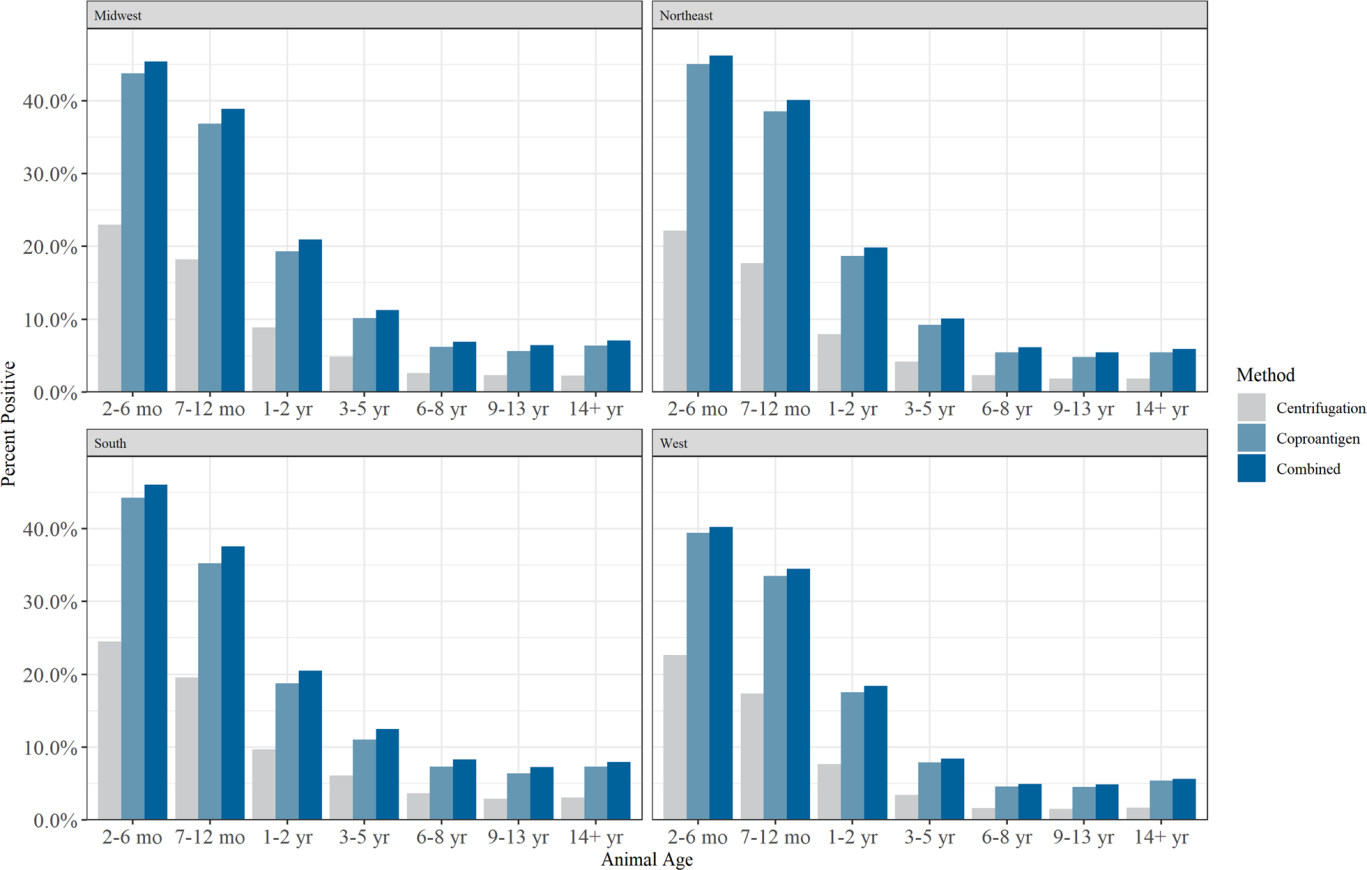
Proportion of test results positive for ascarids, *Giardia*, hookworms or whipworms by the centrifugation method and coproantigen assay according to U.S. Census Bureau regions and age category. Combined refers to positive results by either the coproantigen immunoassay or the centrifugation method

When all fecal test results were combined (including ascarids, *Giardia* spp., hookworms and whipworms), the proportion of positive results varied by geographical region (according to U.S. Census Bureau regions), regardless of veterinary visit type. *Giardia* spp. and *Cystoisospora* spp. infections were the highest in samples from the West (Table 2). Ascarid infections were highest in samples from the Midwest (Table 3). Whipworm infections were the highest in samples from the Midwest and the South (Tables 2, 3). Hookworm infections were the highest in the South (Table 2). Tapeworm infections were highest in the Midwest and the West (Tables 2, 3). When examined by age category and region, samples from younger dogs tended to have higher rates of positivity for hookworms, ascarids and *Giardia* spp. in every region, with a higher number of positive results found by the coproantigen assay (Fig. 4; Additional file 3: Figure S1). Regardless of geographical region, the copraantigen assay was observed to find a higher proportion of positive test results than microscopy for *Giardia*, ascarids, hookworms and whipworms (Fig. 4; all comparisons Pearson Chi-square *P* value < 0.001).

**Table 2:** Proportion of positive test results across all parasites according to the South and West U.S. Census Bureau regions

Parasite	South			West		
	Coproantigen immunoassay (95% CI)	Centrifugation (95% CI)	Combined^a^ (95% CI)	Coproantigen immunoassay (95% CI)	Centrifugation (95% CI)	Combined^a^ (95% CI)
*Giardia*	2.7% (2.6–2.7)	8.8% (8.7–8.9)	9.0% (8.8–9.1)	5.6% (5.5–5.7)	13.3% (13.2–13.5)	13.5% (13.3–13.6)
Hookworm	3.6% (3.6–3.6)	5.6% (5.5–5.6)	6.4% (6.3–6.5)	0.7% (0.6–0.7)	1.0% (1.0–1.1)	1.2% (1.2–1.3)
Ascarid	1.2% (1.2–1.3)	1.4% (1.3–1.4)	1.7% (1.7–1.7)	1.8% (1.7–1.8)	2.0% (2.0–2.1)	2.3% (2.2–2.4)
*Emeria*	1.3% (1.3–1.3)	–	–	1.0% (1.0–1.1)	–	–
*Cystoisospora*	1.0% (1.0–1.0)	–	–	2.7% (2.6–2.8)	–	–
Whipworm	0.9% (0.9–1.0)	1.0% (1.0–1.0)	1.4% (1.3–1.4)	0.3% (0.3–0.3)	0.4% (0.4–0.4)	0.5% (0.5–0.5)
Tapeworm	0.2% (0.2–0.2)	–	–	0.3% (0.3–0.3)	–	–

Values are presented as the percentage test positivity, with the 95% CI in parenthesis

^a^Combined represents positive by either coproantigen or centrifugation

**Table 3:** Proportion of positive test results across all parasites according to the Midwest and Northeast U.S. Census Bureau regions

Parasite	Midwest			Northeast		
	Centrifugation (95% CI)	Coproantigen immunoassay (95% CI)	Combined^a^ (95% CI)	Centrifugation (95% CI)	Coproantigen immunoassay (95% CI)	Combined^a^ (95% CI)
*Giardia*	3.7% (3.6–3.8)	12.1% (11.9–12.3)	12.3% (12.1–12.4)	3.4% (3.3–3.4)	11.5% (11.4–11.6)	11.6% (11.5–11.7)
Hookworm	1.9% (1.8–1.9)	3.1% (3.0–3.1)	3.6% (3.6–3.7)	2.0% (1.9–2.0)	3.2% (3.2–3.3)	3.8% (3.7–3.8)
Ascarid	2.0% (1.9–2.0)	2.2% (2.2–2.3)	2.7% (2.6–2.7)	1.8% (1.7–1.8)	1.9% (1.9–2.0)	2.3% (2.3–2.4)
*Emeria*	1.9% (1.9–1.9)	–	–	2.2% (2.1–2.2)	–	–
*Cystoisospora*	1.4% (1.3–1.4)	–	–	1.6% (1.6–1.6)	–	–
Whipworm	0.8% (0.8–0.9)	1.1% (1.1–1.1)	1.4% (1.4–1.4)	0.7% (0.7–0.7)	0.9% (0.9–1.0)	1.2% (1.1–1.2)
Tapeworm	0.3% (0.3–0.3)	–	–	0.3% (0.3–0.3)	–	–

Values are presented as the percentage test positivity, with the 95% CI in parenthesis

^a^Combined represents positive by either coproantigen or centrifugation

## Discussion

Overall, and regardless of the test method used, the highest proportion of positivity in the canine fecal samples was found for *Giardia* and hookworm parasites. Examination of the age distribution revealed a strong tendency for a higher proportion of intestinal parasite-positive samples in pups. However, the risk of intestinal parasite infections is not limited to pups; year-round, lifelong testing and prevention is recommended for dogs of all ages. Coproantigen testing consistently identified more animals as positive for hookworms, ascarids, whipworms and *Giardia* than centrifugation.

Previous studies have shown regional differences in intestinal parasite infection in the USA [1, 5, 6, 9]. In agreement with these earlier reports [1, 5], our analysis showed that canine fecal samples from the West had the highest* Giardia*-positive rate. *Giardia*-positive results are often seen in dogs presenting to clinics with diarrhea or vomiting and may be expected in shelter dogs and dogs from breeding facilities or kennels [2]. However, recent data support similar *Giardia*-positive findings in client-owned dogs that frequent dog parks, suggesting that subclinical infections may contribute to environmental contamination [1]. In our study, *Cystoisospora* infection was highest in samples from the West, followed by the Northeast, and was lowest in samples from the South; in comparison, Little et al. found the highest *Cystoisospora* infection in the West and Midwest [5], and Blagburn et al. found the highest in the South [9]. While our findings are in partial agreement with those reported by Little at al. [5], they do differ from those of Blagburn et al. [9], with one possible explanation being the difference in canine population: the Blagburn et al. study focused on shelters across the USA and our focus was on samples collected from pet dogs. Compared to both of these earlier studies, we observed a much smaller proportion of positive samples for *Cystoisospora*. In our study, hookworm infection was highest in the South, as was observed in previous studies [1, 5, 9]. As also reported by Little et al. [5] and Stafford et al. [1], we observed that the highest proportion of ascarid-positive samples were from the Midwest [5]. An earlier study reported that the highest proportion of samples positive for whipworm were from the Midwest, South and Northeast [3]. Our findings are in agreement with previous studies that found whipworm infection was identified less in the West [1, 5, 9]. The tendency for pups to have a higher number of tests positive for hookworms, ascarids, whipworms and *Giardia* is present in every region, as is the tendency for more positive results with antigen tests. This age-dependent relationship has been shown previously by Little et al. [5].

Coproantigen testing was observed to have consistently shown a higher proportion of parasite-positive fecal samples compared to centrifugation, regardless of parasite, region or age. Both age-dependent and region-dependent trends in the proportion of parasite-positive samples were observed by each method, with decreasing positivity rates with increasing age of dogs. This similarity in trends of test-positivity suggests that both test methods are representative of the underlying prevalence in these populations. While the coproantigen immunoassay was observed to have a higher proportion of positive test results than the centrifugation method, the true prevalence is not known and cannot be determined from this study.

The authors of previous studies have also concluded that positive antigen results have a high level of specificity. Elsemore et al. tested 100 species-pathogen-free dog fecal samples using the Fecal Dx^®^ immunoassay plate for hookworms, ascarids and whipworms and found a high specificity, with all 100 samples testing negative for all three parasites [21]. High antigen specificity contributes to improved accuracy in cases where spurious eggs due to coprophagy might have otherwise resulted in an incorrect diagnosis if identified by flotation alone. *Eimeria* spp. are pseudoparasites that are not infective to dogs and their presence in canine fecal samples may be a result of coprophagic behavior. The presence of *Eimeria* spp., *Toxocara cati* or large strongyles in a fecal sample may be misinterpreted, and thus lead to unnecessary treatment. In addition, Adolph and colleagues found that utilization of the Fecal Dx® immunoassay in conjunction with zinc sulfate centrifugation increased the sensitivity for the detection of intestinal parasites when using necropsy as the gold standard in a cohort of 97 dogs [22].

There are several potential limitations to the study. Although this study examined samples from a large geographical population, some areas were still under-represented (< 0.1% of the testing data), including Montana (*n* = 576), Wyoming (*n* = 698), Mississippi (*n* = 895), South Dakota (*n* = 1003) and North Dakota (*n* = 1204). In addition, this population consisted of reference laboratory data from a single commercial provider and thus does not represent the prevalence of the true population, but rather the proportion of positive test results from a single reference laboratory provider. Patient history, deworming medication, physical examination findings or other diagnostic tests were not collected. Samples from shelters are known to have a much higher prevalence of intestinal parasites [9] and were not excluded from this study. Dogs that had a second test 180 days past their first submission were included in this study, and positive results were considered to be new infections. Thus, if dogs with a second submission had a higher chance of having a positive result compared to dogs that did not have a second submission, the positivity rates could be biased. Although coinfections made up < 0.1% of positive samples they were not excluded from the overall proportion of positive samples, possibly having a small impact on the results.

## Conclusion

This study found that the percentage of samples which tested positive using the traditional fecal flotation by centrifugation method combined with coproantigen immunoassay testing was higher than the percentage of samples which tested positive using traditional flotation by centrifugation alone, regardless of age, visit type or geographic region. As a result, combining coproantigen immunoassay testing with the flotation by centrifugation for identification of intestinal parasitism in dogs may lead to a higher percentage of positive results, suggesting that parasite prevalence in dogs in the USA may be higher than previous studies have indicated.

## Supplementary Information

**Additional file 1: Table S1. **Proportion of dogs tested using flotation by centrifugation and coproantigen during a Wellness or Other Clinical Visits by age category.

**Additional file 2: Table S2. **Proportion of dogs with a positive test result for an intestinal parasite by centrifugation or coproantigen and binary age group (puppy < 2 years old, adults ≥ 2 years old).

**Additional file 3: Figure S1.**Proportion with a positive test results for flotation by centrifugation and coproantigen by region broken down by U.S. Census Bureau region, parasite and age category. Parasites included: *Giardia*, hookworm, ascarid, whipworm, *Eimeria*, *Cystoisospora* and tapeworm.

**Additional file 4: Table S3.**Proportion of dogs with a positive test result for an intestinal parasite by the centrifugation method or coproantigen immunoassay.

**Additional file 5: Table S4.** Combined co-infection rate by either the centrifugation method or coproantigen immunoassay.

**Additional file 6: Table S5.**Number of positive test results by parasite and method.

**Additional file 7: Figure S2.**Proportion of dogs with a positive test result for any intestinal parasite by either the centrifugation method or coproantigen immunoassay by age category.

**Additional file 8: Figure S3.** Proportion of dogs with positive test results for intestinal parasites by the centrifugation method and coproantigen immunoassay by age category.

**Additional file 9: Table S6.** Proportion of dogs with positive test results for intestinal parasites by the centrifugation method and coproantigen immunoassay by age category.

## Data Availability

All data generated or analyzed during this study are included in this published article [and its supplementary information files].

## Abbreviation

CAPC: Companion Animal Parasite Council
