# Supplementary material for: A 3-year retrospective analysis of canine intestinal parasites: fecal testing positivity by age, U.S. geographical region and reason for veterinary visit
Source: Parasit Vectors. 2021 Mar 20;14:173. doi: 10.1186/s13071-021-04678-6 (PMC7981966; doi:10.1186/s13071-021-04678-6)
Supplement: Supplementary file 1 — Additional file 1: Table S1. Proportion of dogs tested using flotation by centrifugation and coproantigen during a Wellness or Other Clinical Visits by age category. [file 13071_2021_4678_MOESM1_ESM.docx]

**Additional file 1**

Age-group differences by clinical exam type were observed. Pups 2 to 6-months of age comprised 18.5% (n= 228,453) of the wellness exam population, and only 8.9% (n = 59,114) of the other clinical population

**Table S1.** Proportion of dogs tested using flotation by centrifugation and coproantigen during a wellness or other clinical veterinary visits by age category.

| Visit Type | 2-6 mo | 7-12 mo | 1-2 yr | 3-5 yr | 6-8 yr | 9-13 yr | 14+ yr | Total |
| --- | --- | --- | --- | --- | --- | --- | --- | --- |
| Wellness | 228,453 (18.5%) | 29,232 (2.4%) | 102,225 (8.3%) | 266,287 (21.5%) | 240,313 (19.4%) | 310,585 (25.1%) | 59,353 (4.8%) | 1,236,448 (64.9%) |
| Other Clinical  Visit | 59,114 (8.9%) | 41,320 (6.2%) | 63,810 (9.6%) | 131,433 (19.7%) | 115,794 (17.3%) | 200,067 (30%) | 55,866 (8.4%) | 667,404 (35.1%) |
| Total | 287,567 (15.1%) | 70,552 (3.7%) | 166,035 (8.7%) | 397,720 (20.9%) | 356,107 (18.7%) | 510,652 (26.8%) | 115,219 (6.1%) | 1,903,852 |

*Giardia* was the most common parasite identified by centrifugation with a positivity of 3.8%, followed by *Hookworm* (2.2%), Ascarid (1.7%), *Cystoisospora* (1.5%), whipworm (0.7%), and tapeworm (0.3%). *Giardia* and hookworm had the highest percent positive on coproantigen with 11.5% and 3.5% positive results respectively. For each intestinal parasite tested by both diagnostic methods, the proportion of positive tests was significantly higher by coproantigen than by centrifugation (Additional file 2: Table S2, Additional file 4: Table S3). The highest co-infection was ascarid and *Giardia* which occurred in 0.32% of all fecal tests. Additional co-infection information can be found in Additional file 5: Table S4.

**Table S2**. Proportion of dogs with a positive test result for an intestinal parasite by centrifugation or coproantigen and binary age group (puppy < 2 years old, adults ≥ 2 years old).

| Visit Type | Age Group | Method | *Cystoisospora* | *Eimeria* | *Giardia* | Hookworm | Ascarid | Tapeworm | Whipworm |
| --- | --- | --- | --- | --- | --- | --- | --- | --- | --- |
| All | < 2 years | Centrifugation | 4.9  (4.8 - 4.9) | 2.6  (2.5 - 2.6) | 8.8   (8.8 - 8.9) | 4.2   (4.2 - 4.3) | 5.3  (5.2 - 5.3) | 0.4   (0.4 - 0.4) | 1.1  (1.1 - 1.1) |
| All | < 2 years | Coproantigen | --- | --- | 16.3  (16.2 - 16.4) | 6.0   (5.9 - 6.1) | 5.8   (5.7 - 5.9) | --- | 1.7  (1.7 - 1.7) |
| All | ≥ 2 years | Centrifugation | 0.3   (0.3 - 0.3) | 1.3  (1.3 - 1.3) | 0.7  (0.7 - 0.7) | 1.5   (1.5 - 1.5) | 0.3   (0.3 - 0.3) | 0.2   (0.2 - 0.2) | 0.6  (0.6 - 0.6) |
| All | ≥ 2 years | Coproantigen | --- | --- | 1.7   (1.7 - 1.8) | 2.6   (2.6 - 2.6) | 0.4  (0.3 - 0.4) | --- | 0.6  (0.6 - 0.6) |
| Wellness | < 2 years | Centrifugation | 5.0   (5.0 - 5.1) | 2.7  (2.6 - 2.7) | 8.6  (8.5 - 8.7) | 4.3   (4.2 - 4.4) | 5.9   (5.8 - 5.9) | 0.4   (0.4 - 0.4) | 1.0  (0.9 - 1.0) |
| Wellness | < 2 years | Coproantigen | --- | --- | 15.5   (15.3 - 15.6) | 6.1 (6.0 - 6.2) | 6.5  (6.4 - 6.5) | --- | 1.6   (1.5 - 1.6) |
| Wellness | ≥ 2 years | Centrifugation | 0.2   (0.2 - 0.2) | 1.4   (1.4 - 1.4) | 0.5   (0.5 - 0.5) | 1.3  (1.3 - 1.4) | 0.3   (0.3 - 0.3) | 0.2   (0.2 - 0.2) | 0.5  (0.5 - 0.5) |
| Wellness | ≥ 2 years | Coproantigen | --- | --- | 1.3   (1.2 - 1.3) | 2.5   (2.4 - 2.5) | 0.3  (0.3 - 0.3) | --- | 0.5   (0.5 - 0.6) |
| Other Clinical Visit | < 2 years | Centrifugation | 4.5   (4.4 - 4.6) | 2.4   (2.3 - 2.4) | 9.4   (9.2 - 9.5) | 4.0   (3.9 - 4.1) | 4.0   (3.9 - 4.1) | 0.5   (0.5 - 0.5) | 1.5   (1.4 - 1.5) |
| Other Clinical Visit | < 2 years | Coproantigen | --- | --- | 18.1   (17.9 - 18.3) | 5.8  (5.6 - 5.9) | 4.3   (4.2 - 4.4) | --- | 2.0   (1.9 - 2.0) |
| Other Clinical Visit | ≥ 2 years | Centrifugation | 0.4   (0.4 - 0.4) | 1.2  (1.1 - 1.2) | 0.9   (0.9 - 1.0) | 1.7  (1.7 - 1.8) | 0.4   (0.3 - 0.4) | 0.3   (0.2 - 0.3) | 0.7  (0.7 - 0.7) |
| Other Clinical Visit | ≥ 2 years | Coproantigen | --- | --- | 2.6  (2.5 - 2.6) | 2.9   (2.9 - 2.9) | 0.4  (0.4 - 0.4) | --- | 0.8  (0.8 - 0.8) |

**Table S3.** Proportion of dogs with a positive test result for an intestinal parasite by centrifugation or coproantigen.

| Parasite |  | Centrifugation  % (95% CI) |  | Coproantigen  % (95% CI) |
| --- | --- | --- | --- | --- |
| *Giardia* |  | 3.8 (3.8 - 3.9) |  | 11.5 (11.4 -11.5) |
| Hookworm | | 2.2 (2.2 - 2.3) |  | 3.5 (3.5 - 3.6) |
| Ascarid |  | 1.7 (1.7 - 1.7) |  | 1.9 (1.8 - 1.9) |
| *Eimeria* |  | 1.7 (1.6 – 1.7) |  | --- |
| *Cystoisospora* |  | 1.5 (1.5 - 1.6) |  | --- |
| Whipworm |  | 0.7 (0.7 - 0.7) |  | 0.9 (0.9 - 0.9) |
| Tapeworm |  | 0.3 (0.3 - 0.3) |  | --- |

**Table S4.** Combined co-infection rate by either centrifugation or coproantigen.

| **Species** | **Percentage** |
| --- | --- |
| **4 Coinfections** |  |
| Hookworm/Ascarid/Whipworm/*Giardia* | 0.01% |
| **3 Coinfections** |  |
| Hookworm/Ascarid/*Giardia* | 0.06% |
| Hookworm/Ascarid/Whipworm | 0.02% |
| Hookworm/Whipworm/*Giardia* | 0.03% |
| Ascarid/Whipworm/*Giardia* | 0.02% |
| **2 Coinfections** |  |
| Hookworm/*Giardia* | 0.26% |
| Hookworm/Ascarid | 0.13% |
| Hookworm/Whipworm | 0.11% |
| Ascarid/*Giardia* | 0.32% |
| Ascarid/Whipworm | 0.03% |
| Whipworm/*Giardia* | 0.07% |

Of the fecal samples that tested positive for ascarids by either method, 16.3% were positive by centrifugation alone, 24.6% were positive by coproantigen alone, and the remaining 59.1% were positive by both methods. For *Giardia*, the positivity by centrifugation alone was 15.6%, 57.2% by coproantigen alone, and 27.2% by both. For hookworm, the contribution by centrifugation alone was 14.0%, 45.8% by coproantigen alone, and 40.2% by both. For whipworm, the contribution by centrifugation alone was 21.9%, 38.0% by coproantigen alone, and 40.0% by both. Numbers of positive tests by each method are detailed in Additional File 6 Table S5.

**Table S5.** Number of positive test results by parasite and method.

| Parasite | Positives  Either  Method |  | Positives  Both  Methods |  | Positives Centri-  fugation |  | Positives Copro-  antigen |  | Centri-  fugation Only |  | Antigen  Only |
| --- | --- | --- | --- | --- | --- | --- | --- | --- | --- | --- | --- |
| *Giardia* | 110504 |  | 35,256 |  | 36521 |  | 109239 |  | 1,265 |  | 73,983 |
| Hookworm | 78,528 |  | 31,585 |  | 42,555 |  | 67,558 |  | 10,970 |  | 35,973 |
| Ascarid | 42,200 |  | 24,935 |  | 31,822 |  | 35,313 |  | 6,887 |  | 10,378 |
| Whipworm | 22,464 |  | 8,991 |  | 13,919 |  | 17,536 |  | 4,928 |  | 8,545 |

When pooled together, pups aged two to six-months were observed to have the highest proportion positive by either centrifugation or coproantigen (Additional file 3: Figure S1). Samples from two to six-month-old pups were found to have the highest proportion of positive test results for both centrifugation and coproantigen compared to samples from other age categories in *Giardia*, *Cystoisospora*, and ascarids (Additional file 6: Table S5, Additional file 7: Figure S2). Both centrifugation and coproantigen in hookworm and whipworms, had the highest proportion positive in seven to twelve-month old pups (Additional file 7: Figure S2, Additional file 9: Table S6).

**Figure S1.** Proportion of dogs with a positive test result for any intestinal parasite by either centrifugation or coproantigen by age category. Parasites included: *Giardia*, hookworm, ascarid, whipworm, *Eimeria*, *Cystoisospora*, and tapeworm.

**
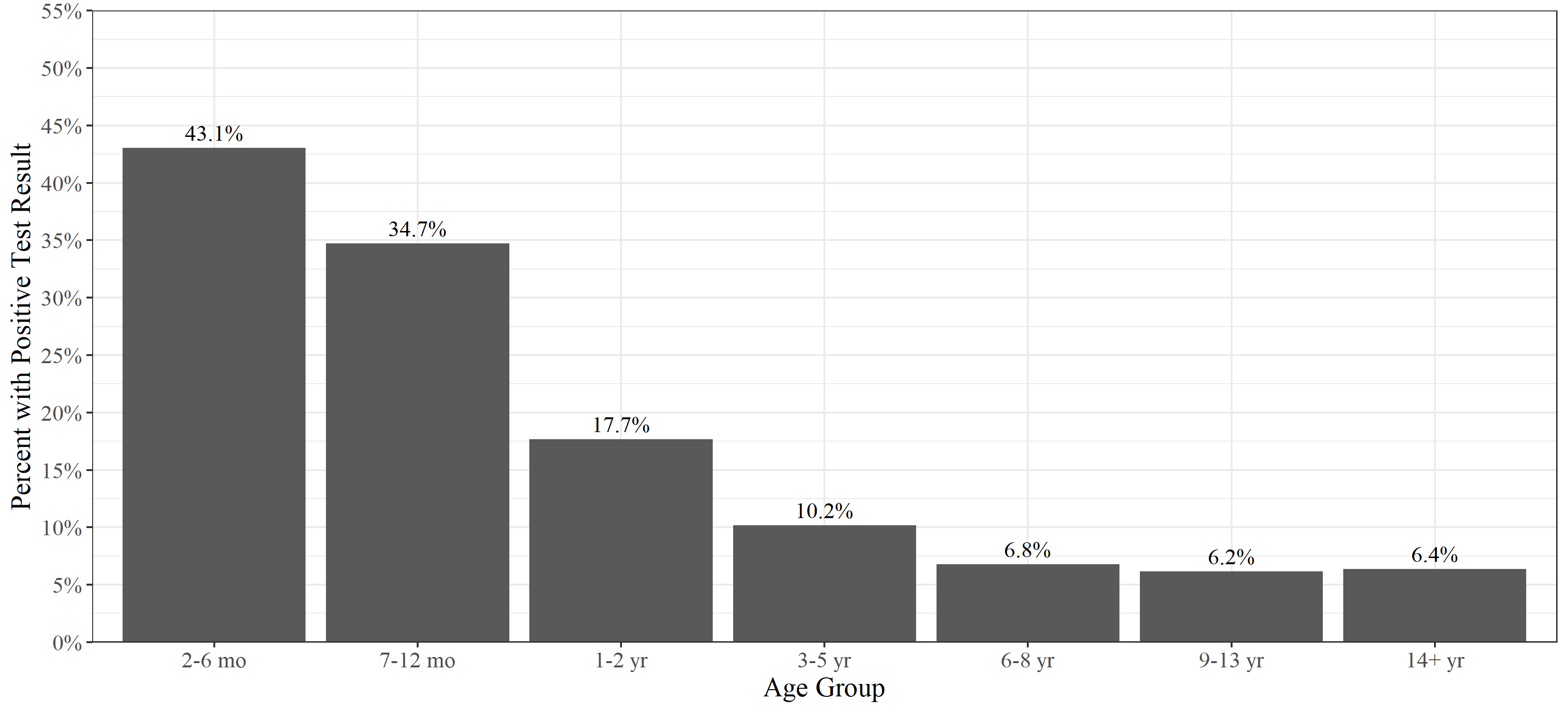
**

**Figure S2.** Proportion of dogs with positive test results for intestinal parasite by centrifugation and coproantigen by age category.

**
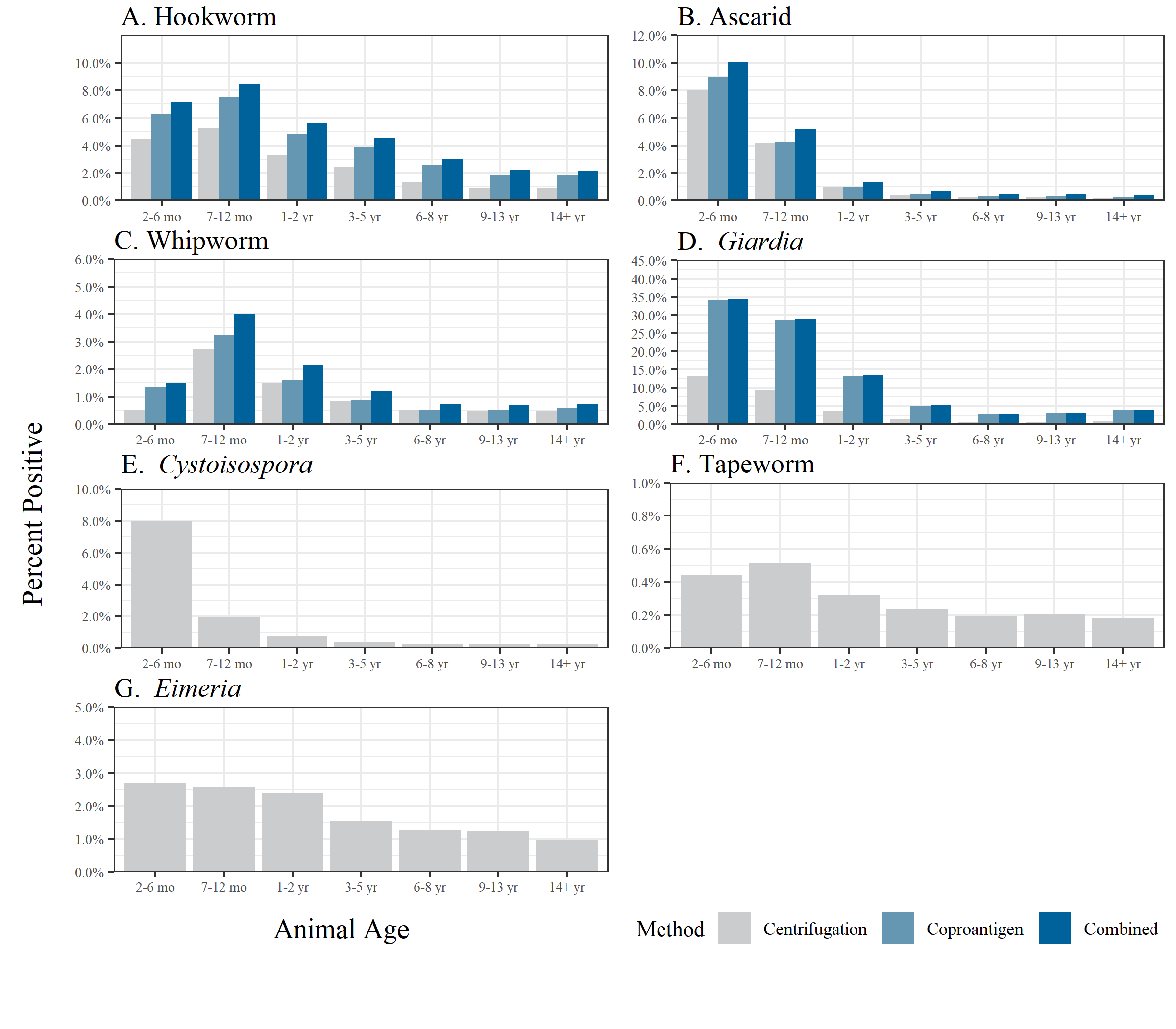
**

**Table S6.** Proportion of dogs with positive test results for intestinal parasite by centrifugation and coproantigen by age category.

| Parasite | Method | 2-6 mo  (95% CI) | 7-12 mo  (95% CI) | 1-2 yr  (95% CI) | 3-5 yr  (95% CI) | 6-8 yr  (95% CI) | 9-13 yr  (95% CI) | 14+ yr  (95% CI) |
| --- | --- | --- | --- | --- | --- | --- | --- | --- |
| Giardia | Centrifugation | 12.3  (12.2 - 12.5) | 8.5  (8.3 - 8.7) | 2.9  (2.8 - 2.9) | 1.0  (0.9 - 1.0) | 0.5  (0.5 - 0.5) | 0.5  (0.5 - 0.6) | 0.8  (0.7 - 0.8) |
| Giardia | Coproantigen | 21.4  (21.2 - 21.5) | 17.9  (17.6 - 18.2) | 6.8  (6.7 - 6.9) | 2.5  (2.4 - 2.5) | 1.3  (1.3 - 1.4) | 1.4  (1.4 - 1.4) | 1.9  (1.8 - 1.9) |
| Hookworm | Centrifugation | 4.5  (4.4 - 4.5) | 5.2  (5.1 - 5.4) | 3.3  (3.2 - 3.4) | 2.4  (2.4 - 2.5) | 1.4  (1.3 - 1.4) | 0.9  (0.9 - 1.0) | 0.9  (0.9 - 1.0) |
| Hookworm | Coproantigen | 6.3  (6.2 - 6.4) | 7.5  (7.3 - 7.7) | 4.8  (4.7 - 4.9) | 3.9  (3.9 - 4.0) | 2.6  (2.5 - 2.6) | 1.8  (1.8 - 1.9) | 1.9  (1.8 - 1.9) |
| Ascarid | Centrifugation | 8.0  (7.9 - 8.1) | 4.2  (4.0 - 4.3) | 1.0  (0.9 - 1.0) | 0.4  (0.4 - 0.5) | 0.3  (0.2 - 0.3) | 0.3  (0.2 - 0.3) | 0.2  (0.2 - 0.2) |
| Ascarid | Coproantigen | 9.0  (8.9 - 9.1) | 4.3  (4.1 - 4.4) | 1.0  (0.9 - 1.0) | 0.5  (0.5 - 0.5) | 0.3  (0.3 - 0.3) | 0.3  (0.3 - 0.3) | 0.3  (0.2 - 0.3) |
| *Eimeria* | Centrifugation | 2.7  (2.6 - 2.8) | 2.6  (2.5 - 2.7) | 2.4  (2.3 - 2.5) | 1.6  (1.5 - 1.6) | 1.3  (1.2 - 1.3) | 1.2  (1.2 - 1.3) | 1.0  (0.9 - 1) |
| *Cystoisospora* | Centrifugation | 7.9  (7.9 - 8.0) | 2.0  (1.9 - 2.1) | 0.8  (0.7 - 0.8) | 0.4  (0.4 - 0.4) | 0.2  (0.2 - 0.2) | 0.2  (0.2 - 0.2) | 0.2  (0.2 - 0.3) |
| Whipworm | Centrifugation | 0.5  (0.5 - 0.5) | 2.7  (2.6 - 2.8) | 1.5  (1.4 - 1.6) | 0.8  (0.8 - 0.9) | 0.5  (0.5 - 0.5) | 0.5  (0.5 - 0.5) | 0.5  (0.4 - 0.5) |
| Whipworm | Coproantigen | 1.4  (1.3 - 1.4) | 3.3  (3.1 - 3.4) | 1.6  (1.5 - 1.7) | 0.9  (0.8 - 0.9) | 0.5  (0.5 - 0.6) | 0.5  (0.5 - 0.5) | 0.6  (0.5 - 0.6) |
| Tapeworm | Centrifugation | 0.4  (0.4 - 0.5) | 0.5  (0.5 - 0.6) | 0.3  (0.3 - 0.3) | 0.2  (0.2 - 0.3) | 0.2  (0.2 - 0.2) | 0.2  (0.2 - 0.2) | 0.2  (0.2 - 0.2) |
