# Supplementary material for: A 3-year retrospective analysis of canine intestinal parasites: fecal testing positivity by age, U.S. geographical region and reason for veterinary visit
Source: Parasit Vectors. 2021 Mar 20;14:173. doi: 10.1186/s13071-021-04678-6 (PMC7981966; doi:10.1186/s13071-021-04678-6)
Supplement: Supplementary file 2 — Additional file 2: Table S2. Proportion of dogs with a positive test result for an intestinal parasite by centrifugation or coproantigen and binary age group (puppy < 2 years old, adults ≥ 2 years old). [file 13071_2021_4678_MOESM2_ESM.docx]

**Additional file 2: Table S2**. Proportion of dogs with a positive test result for an intestinal parasite by centrifugation or coproantigen and binary age group (puppy < 2 years old, adults ≥ 2 years old).

| Visit Type | Age Group | Method | *Cystoisospora* | *Eimeria* | *Giardia* | Hookworm | Ascarid | Tapeworm | Whipworm |
| --- | --- | --- | --- | --- | --- | --- | --- | --- | --- |
| All | < 2 years | Centrifugation | 4.9  (4.8 - 4.9) | 2.6  (2.5 - 2.6) | 8.8   (8.8 - 8.9) | 4.2   (4.2 - 4.3) | 5.3  (5.2 - 5.3) | 0.4   (0.4 - 0.4) | 1.1  (1.1 - 1.1) |
| All | < 2 years | Coproantigen | --- | --- | 16.3  (16.2 - 16.4) | 6.0   (5.9 - 6.1) | 5.8   (5.7 - 5.9) | --- | 1.7  (1.7 - 1.7) |
| All | ≥ 2 years | Centrifugation | 0.3   (0.3 - 0.3) | 1.3  (1.3 - 1.3) | 0.7  (0.7 - 0.7) | 1.5   (1.5 - 1.5) | 0.3   (0.3 - 0.3) | 0.2   (0.2 - 0.2) | 0.6  (0.6 - 0.6) |
| All | ≥ 2 years | Coproantigen | --- | --- | 1.7   (1.7 - 1.8) | 2.6   (2.6 - 2.6) | 0.4  (0.3 - 0.4) | --- | 0.6  (0.6 - 0.6) |
| Wellness | < 2 years | Centrifugation | 5.0   (5.0 - 5.1) | 2.7  (2.6 - 2.7) | 8.6  (8.5 - 8.7) | 4.3   (4.2 - 4.4) | 5.9   (5.8 - 5.9) | 0.4   (0.4 - 0.4) | 1.0  (0.9 - 1.0) |
| Wellness | < 2 years | Coproantigen | --- | --- | 15.5   (15.3 - 15.6) | 6.1 (6.0 - 6.2) | 6.5  (6.4 - 6.5) | --- | 1.6   (1.5 - 1.6) |
| Wellness | ≥ 2 years | Centrifugation | 0.2   (0.2 - 0.2) | 1.4   (1.4 - 1.4) | 0.5   (0.5 - 0.5) | 1.3  (1.3 - 1.4) | 0.3   (0.3 - 0.3) | 0.2   (0.2 - 0.2) | 0.5  (0.5 - 0.5) |
| Wellness | ≥ 2 years | Coproantigen | --- | --- | 1.3   (1.2 - 1.3) | 2.5   (2.4 - 2.5) | 0.3  (0.3 - 0.3) | --- | 0.5   (0.5 - 0.6) |
| Other Clinical Visit | < 2 years | Centrifugation | 4.5   (4.4 - 4.6) | 2.4   (2.3 - 2.4) | 9.4   (9.2 - 9.5) | 4.0   (3.9 - 4.1) | 4.0   (3.9 - 4.1) | 0.5   (0.5 - 0.5) | 1.5   (1.4 - 1.5) |
| Other Clinical Visit | < 2 years | Coproantigen | --- | --- | 18.1   (17.9 - 18.3) | 5.8  (5.6 - 5.9) | 4.3   (4.2 - 4.4) | --- | 2.0   (1.9 - 2.0) |
| Other Clinical Visit | ≥ 2 years | Centrifugation | 0.4   (0.4 - 0.4) | 1.2  (1.1 - 1.2) | 0.9   (0.9 - 1.0) | 1.7  (1.7 - 1.8) | 0.4   (0.3 - 0.4) | 0.3   (0.2 - 0.3) | 0.7  (0.7 - 0.7) |
| Other Clinical Visit | ≥ 2 years | Coproantigen | --- | --- | 2.6  (2.5 - 2.6) | 2.9   (2.9 - 2.9) | 0.4  (0.4 - 0.4) | --- | 0.8  (0.8 - 0.8) |
