# Supplementary material for: A 3-year retrospective analysis of canine intestinal parasites: fecal testing positivity by age, U.S. geographical region and reason for veterinary visit
Source: Parasit Vectors. 2021 Mar 20;14:173. doi: 10.1186/s13071-021-04678-6 (PMC7981966; doi:10.1186/s13071-021-04678-6)
Supplement: Supplementary file 3 — Additional file 3: Figure S1.Proportion with a positive test results for flotation by centrifugation and coproantigen by region broken down by U.S. Census Bureau region, parasite and age category. Parasites included: Giardia, hookworm, ascarid, whipworm, Eimeria, Cystoisospora and tapeworm. [file 13071_2021_4678_MOESM3_ESM.docx]

**Additional file 3: Figure S1.** Proportion of dogs with a positive test result for any intestinal parasite by either centrifugation or coproantigen by age category. Parasites included: *Giardia*, hookworm, ascarid, whipworm, *Eimeria*, *Cystoisospora*, and tapeworm.

**
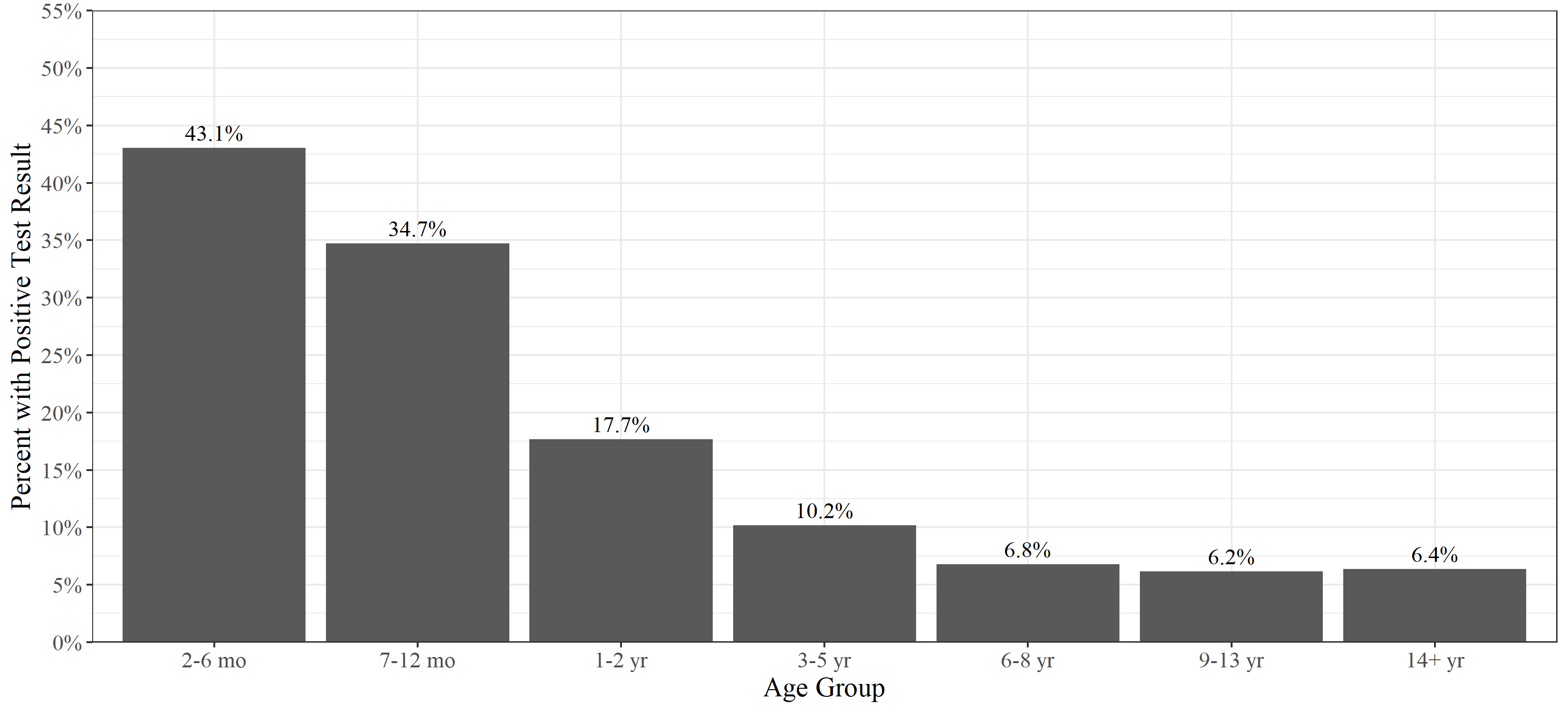
**
