# Supplementary material for: A 3-year retrospective analysis of canine intestinal parasites: fecal testing positivity by age, U.S. geographical region and reason for veterinary visit
Source: Parasit Vectors. 2021 Mar 20;14:173. doi: 10.1186/s13071-021-04678-6 (PMC7981966; doi:10.1186/s13071-021-04678-6)
Supplement: Supplementary file 4 — Additional file 4: Table S3.Proportion of dogs with a positive test result for an intestinal parasite by the centrifugation method or coproantigen immunoassay. [file 13071_2021_4678_MOESM4_ESM.docx]

**Additional file 4: Table S3.** Proportion of dogs with a positive test result for an intestinal parasite by centrifugation or coproantigen.

| Parasite |  | Centrifugation  % (95% CI) |  | Coproantigen  % (95% CI) |
| --- | --- | --- | --- | --- |
| *Giardia* |  | 3.8 (3.8 - 3.9) |  | 11.5 (11.4 -11.5) |
| Hookworm | | 2.2 (2.2 - 2.3) |  | 3.5 (3.5 - 3.6) |
| Ascarid |  | 1.7 (1.7 - 1.7) |  | 1.9 (1.8 - 1.9) |
| *Eimeria* |  | 1.7 (1.6 – 1.7) |  | --- |
| *Cystoisospora* |  | 1.5 (1.5 - 1.6) |  | --- |
| Whipworm |  | 0.7 (0.7 - 0.7) |  | 0.9 (0.9 - 0.9) |
| Tapeworm |  | 0.3 (0.3 - 0.3) |  | --- |
