# Supplementary material for: A 3-year retrospective analysis of canine intestinal parasites: fecal testing positivity by age, U.S. geographical region and reason for veterinary visit
Source: Parasit Vectors. 2021 Mar 20;14:173. doi: 10.1186/s13071-021-04678-6 (PMC7981966; doi:10.1186/s13071-021-04678-6)
Supplement: Supplementary file 5 — Additional file 5: Table S4. Combined co-infection rate by either the centrifugation method or coproantigen immunoassay. [file 13071_2021_4678_MOESM5_ESM.docx]

**Additional file 5: Table S4.** Combined co-infection rate by either centrifugation or coproantigen.

| **Species** | **Percentage** |
| --- | --- |
| **4 Coinfections** |  |
| Hookworm/Ascarid/Whipworm/*Giardia* | 0.01% |
| **3 Coinfections** |  |
| Hookworm/Ascarid/*Giardia* | 0.06% |
| Hookworm/Ascarid/Whipworm | 0.02% |
| Hookworm/Whipworm/*Giardia* | 0.03% |
| Ascarid/Whipworm/*Giardia* | 0.02% |
| **2 Coinfections** |  |
| Hookworm/*Giardia* | 0.26% |
| Hookworm/Ascarid | 0.13% |
| Hookworm/Whipworm | 0.11% |
| Ascarid/*Giardia* | 0.32% |
| Ascarid/Whipworm | 0.03% |
| Whipworm/*Giardia* | 0.07% |

Of the fecal samples that tested positive for ascarids by either method, 16.3% were positive by centrifugation alone, 24.6% were positive by coproantigen alone, and the remaining 59.1% were positive by both methods. For *Giardia*, the positivity by centrifugation alone was 15.6%, 57.2% by coproantigen alone, and 27.2% by both. For hookworm, the contribution by centrifugation alone was 14.0%, 45.8% by coproantigen alone, and 40.2% by both. For whipworm, the contribution by centrifugation alone was 21.9%, 38.0% by coproantigen alone, and 40.0% by both. Numbers of positive tests by each method are detailed in Additional File 6 Table S5.
