# Supplementary material for: A 3-year retrospective analysis of canine intestinal parasites: fecal testing positivity by age, U.S. geographical region and reason for veterinary visit
Source: Parasit Vectors. 2021 Mar 20;14:173. doi: 10.1186/s13071-021-04678-6 (PMC7981966; doi:10.1186/s13071-021-04678-6)
Supplement: Supplementary file 6 — Additional file 6: Table S5.Number of positive test results by parasite and method. [file 13071_2021_4678_MOESM6_ESM.docx]

**Additional file 6: Table S5.** Number of positive test results by parasite and method.

| Parasite | Positives  Either  Method |  | Positives  Both  Methods |  | Positives Centri-  fugation |  | Positives Copro-  antigen |  | Centri-  fugation Only |  | Antigen  Only |
| --- | --- | --- | --- | --- | --- | --- | --- | --- | --- | --- | --- |
| *Giardia* | 110504 |  | 35,256 |  | 36521 |  | 109239 |  | 1,265 |  | 73,983 |
| Hookworm | 78,528 |  | 31,585 |  | 42,555 |  | 67,558 |  | 10,970 |  | 35,973 |
| Ascarid | 42,200 |  | 24,935 |  | 31,822 |  | 35,313 |  | 6,887 |  | 10,378 |
| Whipworm | 22,464 |  | 8,991 |  | 13,919 |  | 17,536 |  | 4,928 |  | 8,545 |

When pooled together, pups aged two to six-months were observed to have the highest proportion positive by either centrifugation or coproantigen (Additional file 3: Figure S1). Samples from two to six-month-old pups were found to have the highest proportion of positive test results for both centrifugation and coproantigen compared to samples from other age categories in *Giardia*, *Cystoisospora*, and ascarids (Additional file 6: Table S5, Additional file 7: Figure S2). Both centrifugation and coproantigen in hookworm and whipworms, had the highest proportion positive in seven to twelve-month old pups (Additional file 7: Figure S2, Additional file 9: Table S6).
