# Supplementary material for: A 3-year retrospective analysis of canine intestinal parasites: fecal testing positivity by age, U.S. geographical region and reason for veterinary visit
Source: Parasit Vectors. 2021 Mar 20;14:173. doi: 10.1186/s13071-021-04678-6 (PMC7981966; doi:10.1186/s13071-021-04678-6)
Supplement: Supplementary file 7 — Additional file 7: Figure S2.Proportion of dogs with a positive test result for any intestinal parasite by either the centrifugation method or coproantigen immunoassay by age category. [file 13071_2021_4678_MOESM7_ESM.docx]

**Additional file 7: Figure S2.** Proportion of dogs with positive test results for intestinal parasite by centrifugation and coproantigen by age category.

**
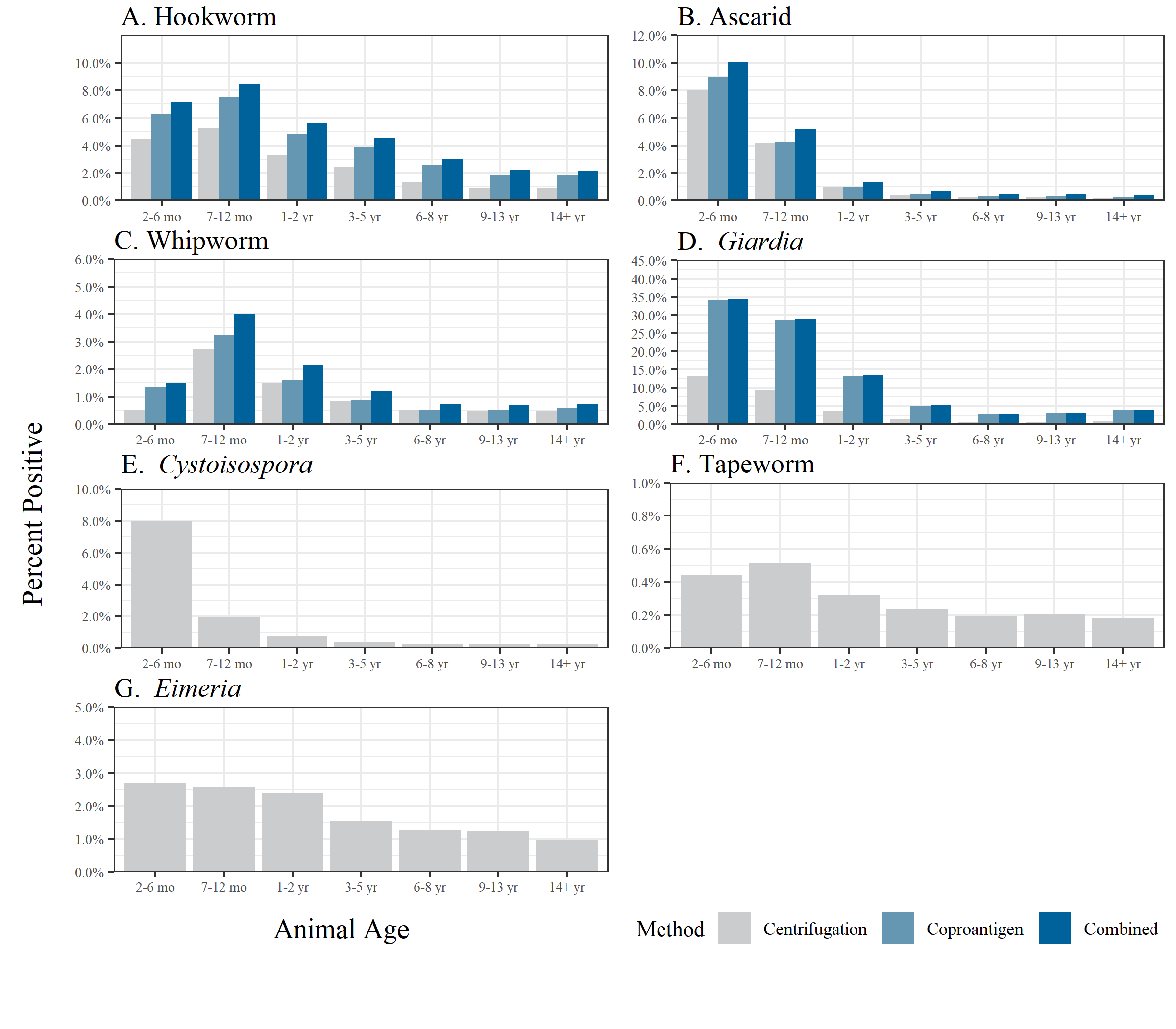
**
