# Supplementary material for: A 3-year retrospective analysis of canine intestinal parasites: fecal testing positivity by age, U.S. geographical region and reason for veterinary visit
Source: Parasit Vectors. 2021 Mar 20;14:173. doi: 10.1186/s13071-021-04678-6 (PMC7981966; doi:10.1186/s13071-021-04678-6)
Supplement: Supplementary file 8 — Additional file 8: Figure S3. Proportion of dogs with positive test results for intestinal parasites by the centrifugation method and coproantigen immunoassay by age category. [file 13071_2021_4678_MOESM8_ESM.docx]

**Additional file 9: Table S6.** Proportion of dogs with positive test results for intestinal parasite by centrifugation and coproantigen by age category.

| Parasite | Method | 2-6 mo  (95% CI) | 7-12 mo  (95% CI) | 1-2 yr  (95% CI) | 3-5 yr  (95% CI) | 6-8 yr  (95% CI) | 9-13 yr  (95% CI) | 14+ yr  (95% CI) |
| --- | --- | --- | --- | --- | --- | --- | --- | --- |
| Giardia | Centrifugation | 12.3  (12.2 - 12.5) | 8.5  (8.3 - 8.7) | 2.9  (2.8 - 2.9) | 1.0  (0.9 - 1.0) | 0.5  (0.5 - 0.5) | 0.5  (0.5 - 0.6) | 0.8  (0.7 - 0.8) |
| Giardia | Coproantigen | 21.4  (21.2 - 21.5) | 17.9  (17.6 - 18.2) | 6.8  (6.7 - 6.9) | 2.5  (2.4 - 2.5) | 1.3  (1.3 - 1.4) | 1.4  (1.4 - 1.4) | 1.9  (1.8 - 1.9) |
| Hookworm | Centrifugation | 4.5  (4.4 - 4.5) | 5.2  (5.1 - 5.4) | 3.3  (3.2 - 3.4) | 2.4  (2.4 - 2.5) | 1.4  (1.3 - 1.4) | 0.9  (0.9 - 1.0) | 0.9  (0.9 - 1.0) |
| Hookworm | Coproantigen | 6.3  (6.2 - 6.4) | 7.5  (7.3 - 7.7) | 4.8  (4.7 - 4.9) | 3.9  (3.9 - 4.0) | 2.6  (2.5 - 2.6) | 1.8  (1.8 - 1.9) | 1.9  (1.8 - 1.9) |
| Ascarid | Centrifugation | 8.0  (7.9 - 8.1) | 4.2  (4.0 - 4.3) | 1.0  (0.9 - 1.0) | 0.4  (0.4 - 0.5) | 0.3  (0.2 - 0.3) | 0.3  (0.2 - 0.3) | 0.2  (0.2 - 0.2) |
| Ascarid | Coproantigen | 9.0  (8.9 - 9.1) | 4.3  (4.1 - 4.4) | 1.0  (0.9 - 1.0) | 0.5  (0.5 - 0.5) | 0.3  (0.3 - 0.3) | 0.3  (0.3 - 0.3) | 0.3  (0.2 - 0.3) |
| *Eimeria* | Centrifugation | 2.7  (2.6 - 2.8) | 2.6  (2.5 - 2.7) | 2.4  (2.3 - 2.5) | 1.6  (1.5 - 1.6) | 1.3  (1.2 - 1.3) | 1.2  (1.2 - 1.3) | 1.0  (0.9 - 1) |
| *Cystoisospora* | Centrifugation | 7.9  (7.9 - 8.0) | 2.0  (1.9 - 2.1) | 0.8  (0.7 - 0.8) | 0.4  (0.4 - 0.4) | 0.2  (0.2 - 0.2) | 0.2  (0.2 - 0.2) | 0.2  (0.2 - 0.3) |
| Whipworm | Centrifugation | 0.5  (0.5 - 0.5) | 2.7  (2.6 - 2.8) | 1.5  (1.4 - 1.6) | 0.8  (0.8 - 0.9) | 0.5  (0.5 - 0.5) | 0.5  (0.5 - 0.5) | 0.5  (0.4 - 0.5) |
| Whipworm | Coproantigen | 1.4  (1.3 - 1.4) | 3.3  (3.1 - 3.4) | 1.6  (1.5 - 1.7) | 0.9  (0.8 - 0.9) | 0.5  (0.5 - 0.6) | 0.5  (0.5 - 0.5) | 0.6  (0.5 - 0.6) |
| Tapeworm | Centrifugation | 0.4  (0.4 - 0.5) | 0.5  (0.5 - 0.6) | 0.3  (0.3 - 0.3) | 0.2  (0.2 - 0.3) | 0.2  (0.2 - 0.2) | 0.2  (0.2 - 0.2) | 0.2  (0.2 - 0.2) |
