# Supplementary figures and images for: A 3-year retrospective analysis of canine intestinal parasites: fecal testing positivity by age, U.S. geographical region and reason for veterinary visit
Source: Parasit Vectors. 2021 Mar 20;14:173. doi: 10.1186/s13071-021-04678-6 (PMC7981966; doi:10.1186/s13071-021-04678-6)

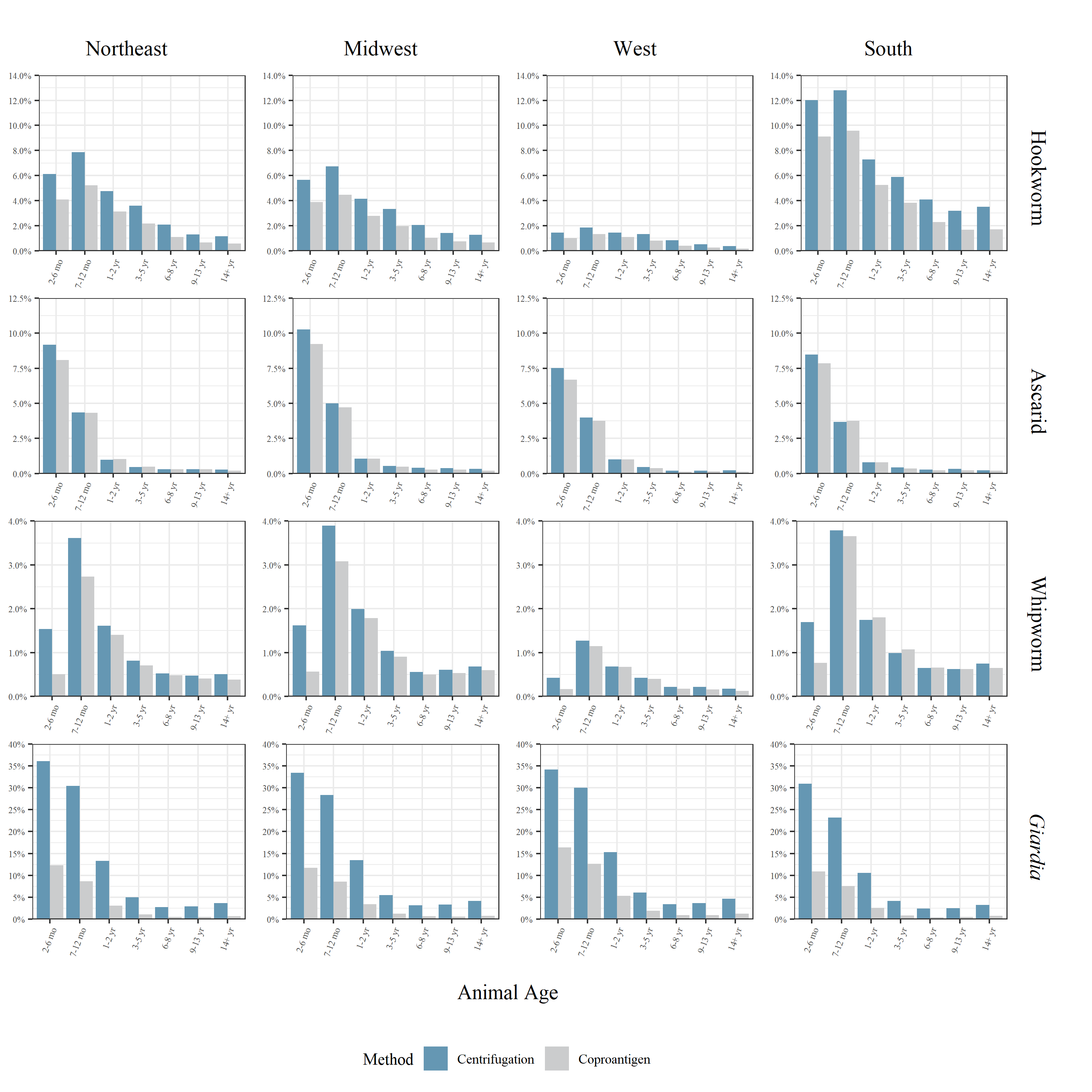

Supplement: Supplementary file 9 — Additional file 9: Table S6. Proportion of dogs with positive test results for intestinal parasites by the centrifugation method and coproantigen immunoassay by age category. [file 13071_2021_4678_MOESM9_ESM.png]
